# Identifying Opportunity Targets in Gram-Negative Pathogens for Infectious Disease Mitigation

**DOI:** 10.1021/acscentsci.4c01437

**Published:** 2025-01-03

**Authors:** Isaac A. Paddy, Laura M. K. Dassama

**Affiliations:** † Department of Chemical and Systems Biology, Stanford School of Medicine, Stanford, California 94305-6104, United States; ‡ Sarafan ChEM-H Institute, Stanford University, Stanford, California 94305-6104, United States; § Department of Chemistry, Stanford University, Stanford, California 94305-6104, United States; ∥ Department Microbiology & Immunology, Stanford School of Medicine, Stanford, California 94305-6104, United States

## Abstract

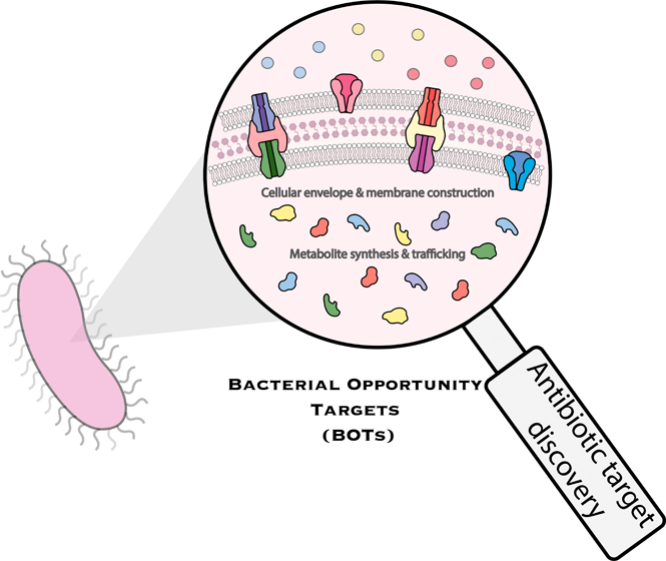

Antimicrobial drug resistance (AMR) is a pressing global human health challenge. Humans face one of their grandest challenges as climate change expands the habitat of vectors that bear human pathogens, incidences of nosocomial infections rise, and new antibiotics discovery lags. AMR is a multifaceted problem that requires a multidisciplinary and an “all-hands-on-deck” approach. As chemical microbiologists, we are well positioned to understand the complexities of AMR while seeing opportunities for tackling the challenge. In this Outlook, we focus on vulnerabilities of human pathogens and posit that they represent “opportunity targets” for which few modulatory ligands exist. We center our attention on proteins in Gram-negative organisms, which are recalcitrant to many antibiotics because of their external membrane barrier. Our hope is to highlight such targets and explore their potential as “druggable” proteins for infectious disease mitigation. We posit that success in this endeavor will introduce new classes of antibiotics that might alleviate some of the current pressing AMR concerns.

## Abstract 2

Antibiotic resistance is rapidly increasing while discovery of new drugs lags. Uncovering of new druggable targets could increase the number of antibiotics and temporarily abate the AMR crisis.

## 1 Introduction

In September 1928, an unexpected discovery in Alexander Fleming’s laboratory marked a pivotal moment in medical history. Returning from a holiday, Fleming noticed something unusual on a Petri dish of *Staphylococcus* bacteria he had left uncovered.^1^ A patch of mold had grown on the plate, but the bacterial colonies were shielded away as if the mold had created a protective barrier. Fleming later identified the mold as *Penicillium notatum* and found that it was releasing a substance capable of killing the bacteria.^1^ This substance, later named penicillin, would revolutionize medicine and introduce the age of antibiotics. The excitement of this breakthrough was quickly tempered by a growing challenge. By the early 1940s (as penicillin was being widely used), scientists observed the emergence of bacterial resistance to the drug. Specific strains of *Staphylococcus aureus* in hospitals showed mutations that rendered them immune to penicillin’s effects.^2,3^As researchers developed new derivatives of penicillin to combat these resistant strains, bacteria quickly adapted and resisted each new drug. This interplay between scientific advancement and bacterial adaptation set the stage for an ongoing struggle and highlights both the importance of antibiotics and the relentless nature of bacterial resistance.

Today, antimicrobial resistance (AMR) continues to be a growing and urgent public health threat. Based on predictive statistical models, there was an estimated 4.85 million AMR-associated deaths in 2019.^4^ The six leading pathogens associated with these deaths are known as the ESKAPE pathogens (**E***nterococcus faecium*, **S***taphylococcus aureus*, **K***lebsiella pneumoniae*, **A***cinetobacter baumannii*, **P***seudomonas aeruginosa* and **E***nterobacter spp.)*.^5,6^ There are also emerging pathogens that are considered high priority by the World Health Organization (WHO). These include *Helicobacter pylori, Campylobacter spp., and Salmonellae*. Compounding the AMR crisis is the fact that we are currently in a discovery void. This void describes the period from 1987 to present, in which there have been no new class of antibiotics successfully used in the clinic.^7,8^Despite intense efforts, we still face the conundrum of developing new classes of antibiotics while keeping up with rapid resistance.

In this Outlook, we highlight recent advances in the development of new molecules that target essential metabolic pathways in diderm or Gram-negative bacteria. We also discuss emerging methods for target identification and explore interesting pathogens as case studies. Through these efforts, we hope to shine light on the importance of filling the discovery void through the inhibition of opportunity targets. We also hope to highlight the immense impact of AMR-associated diseases on under-resourced countries and marginalized communities around the world.

## 2 Understanding Bacterial Pathogens with Limited Metabolic Capabilities and Modes of Resistance

Bacterial pathogens with limited metabolic capabilities contain a reduced set of biosynthetic pathways that restrict their ability to synthesize essential biomolecules. These organisms rely heavily on their host for nutrients, which makes them difficult to culture in vitro and limits our understanding of their biology. It is not unusual to find that these bacteria have sometimes lost genes associated with the synthesis of amino acids, nucleotides, and other vital metabolites and molecules. An example is *Mycoplasmas*, where species like *Mycoplasma pneumoniae* lack many of the genes required for peptidoglycan (PG) synthesis, thereby making them naturally resistant to antibiotics that target PG biosynthesis.^9^ This metabolic minimalism is not merely a consequence of adaptation but is also a strategy that allows pathogens to evade detection by the host immune system and avoid inhibition by certain classes of antibiotics.

Infections caused by these pathogens often result in chronic conditions characterized by persistent, low-level symptoms that are difficult to diagnose and treat. *Chlamydia trachomatis*, a pathogen with a reduced genome and limited metabolic pathways, is a key example as it is been known to cause long-lasting infections that are associated with serious complications that include pelvic inflammatory disease and infertility.^10−12^ The reliance of these bacteria on the host for nutrients makes them proficient at surviving within host cells where they can evade the immune system and many antibiotics. Their intracellular nature further complicates treatment because many antibiotics cannot effectively penetrate host cells to reach the bacteria in sufficient concentrations. As a result, the standard treatment regimens are often prolonged, and the risk of treatment failure and recurrence is high.^13^ The clinical challenges posed by these pathogens highlight the urgent need for more effective diagnostic tools and therapeutic approaches.

Gram-negative bacteria in particular pose a particularly challenging feat for not only treatment of infection, but also diagnosis and vaccination. Many Gram-negative bacteria in the context of host–pathogen interaction use a myriad of approaches to evade the immune system and survive in many organs throughout the body. These include employing intrinsic characteristics such as mimicking the membrane composition of eukaryotic cells to evade immune responses^14^ and more extrinsic factors such as the release of proteins to prevent detection by host immune factors.^15^

**Figure 1:**
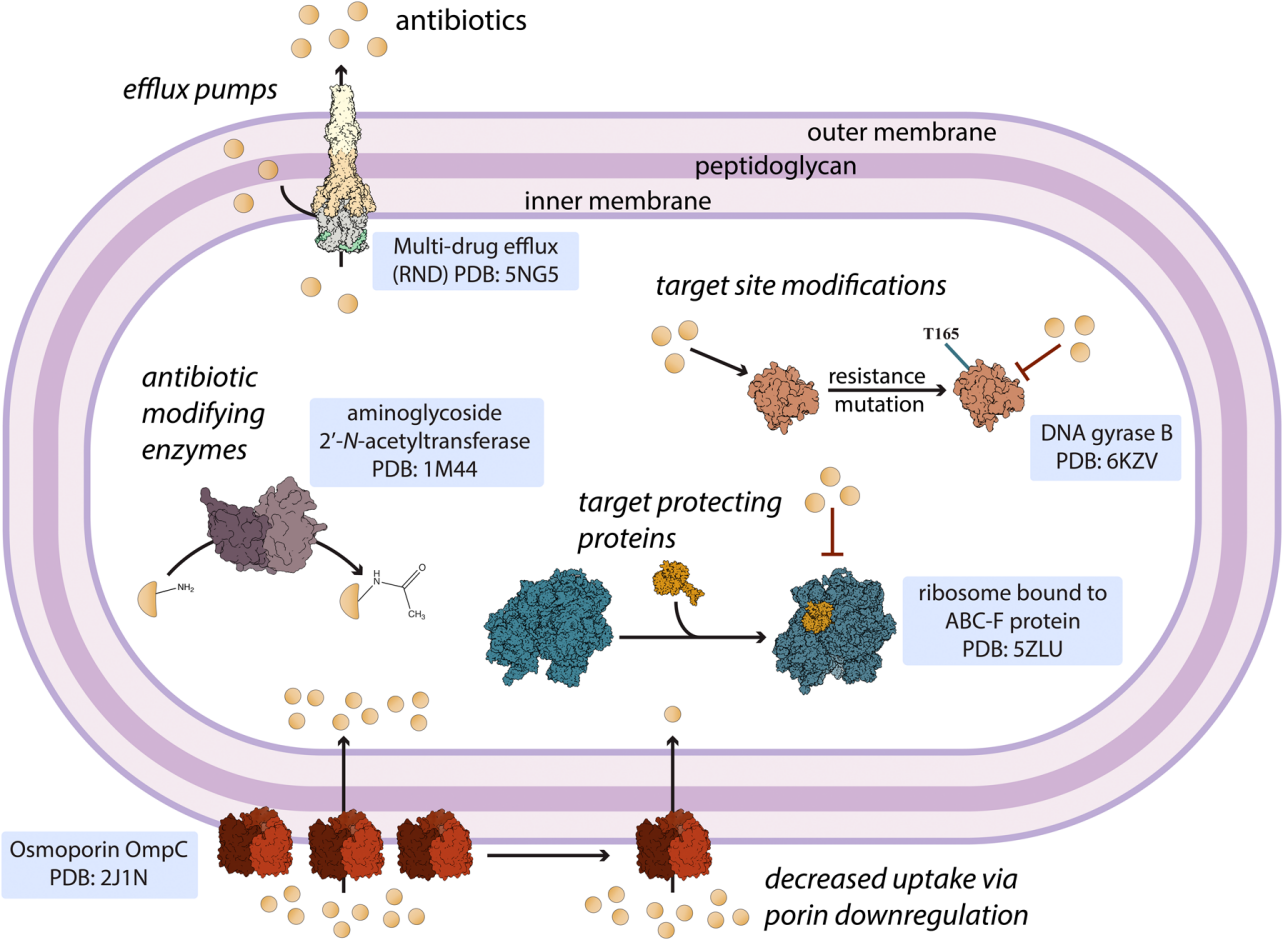
An overview of antibiotic resistance pathways in bacteria that highlights examples of proteins and enzymes involved in the key resistance mechanisms. These include drug efflux, antibiotic modification, target alteration, target protecting proteins, and decreased drug uptake with porins.

These specialized metabolic adaptations underscore the need for research into the unique biology of pathogens to uncover vulnerabilities that can be exploited and targeted for therapeutic purposes. Many bacteria use widely recognized resistance mechanisms that include enzymatic degradation of antibiotics, modification of drug targets, active efflux systems, and reduced membrane permeability (Figure 1). A new age of target discovery calls for the ability to 1) understand the intricate nature of bacterial resistance pathways and their plasticity and 2) develop and test new chemical matter that cannot be evaded using these mechanisms and therefore have a low probability of bacterial resistance. This Outlook will therefore highlight the recent discovery of targets that are essential to many Gram-negative bacteria, and the discovery of drugs that exploit them.

## 3 Cellular Envelope and Membrane Construction

### 3.1 Lipopolysaccharide (LPS)

**Figure 2:**
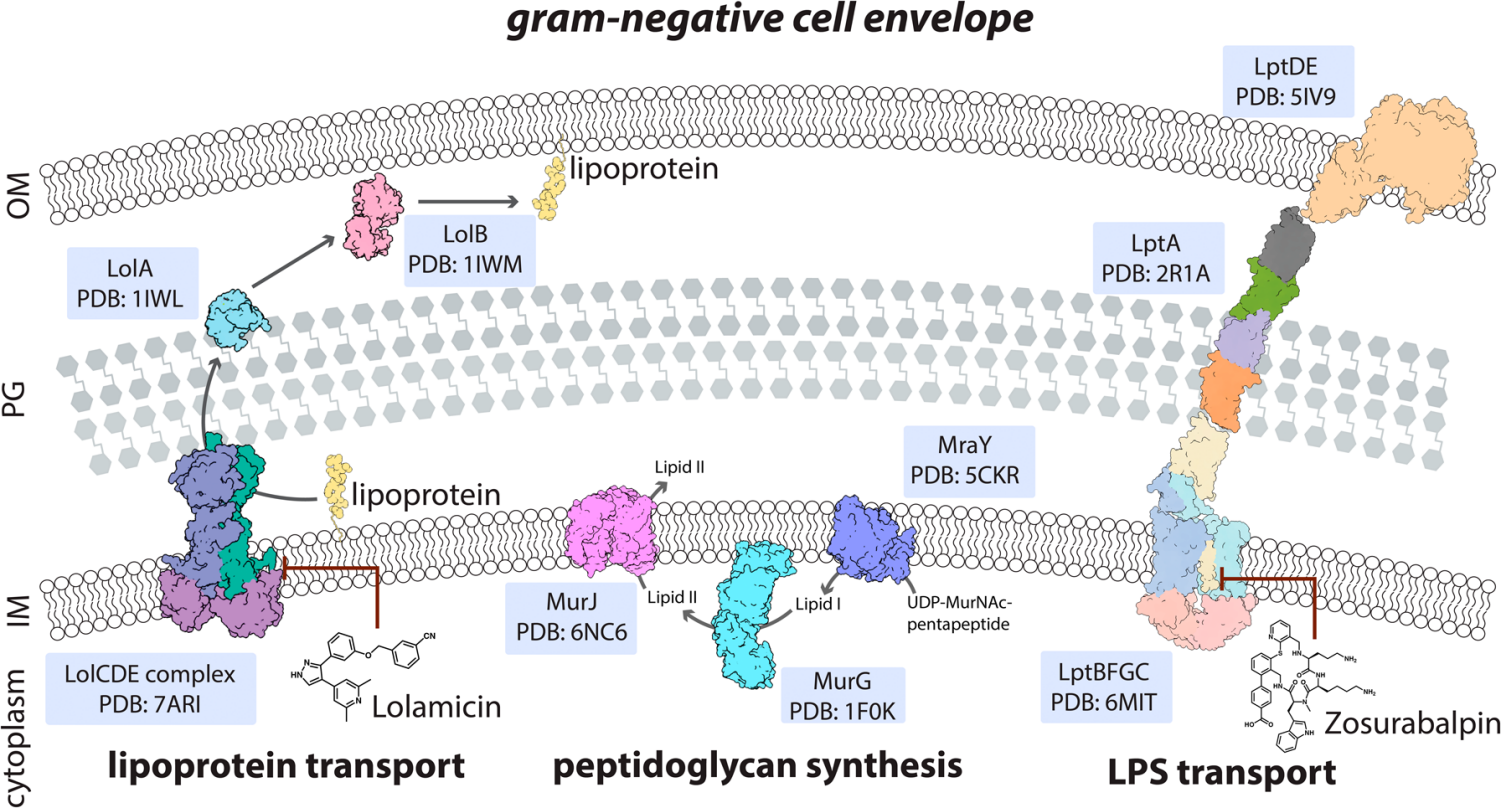
Bacterial cellular envelop construction pathways. Lipoprotein transport involves action of the Lol proteins (Lolamicin is a novel antibiotic that selectively inhibits the LolCDE complex). Key players in peptidoglycan assembly are highlighted in the middle. The LPS transport pathway shown to highlight the proteins that shuttle LPS from the inner to outer membrane. Highlighted is Zosurabalpin, which selectively targets LPS transport via the Lpt proteins.

LPS is an essential component of the outer membrane of most Gram-negative bacterial cell envelopes. The glycoconjugate provides structural integrity to cells and offers protection by acting as a barrier against harmful substances that include antibiotics and other environmental challenges.^16,17^ LPS is structurally diverse and can act as an immunogen that allows host cells to detect and respond to bacterial pathogens.^17−19^ With this, it has been proposed that LPS and the LPS-binding protein (LBP) can be used as biomarkers, as bacteria make unique forms of LPS.^20−22^ Bacteria rely on a streamlined set of proteins known as the lipopolysaccharide transport (Lpt) system to assemble and transport LPS to the outer membrane (Figure 2).^23,24^ The Lpt system is essential for the survival of many Gram-negative pathogens,^25,26^ and targeting this system has become a promising strategy to combat AMR because most bacteria lack compensatory mechanisms for LPS loss.^27−30^ The recent discovery of inhibitors like Zosurabalpin, a tethered macrocyclic peptide that targets the inner membrane LptB_2_FGC complex in carbapenem-resistant *Acinetobacter baumannii*, shows promise that novel targets and antibiotic modes of action can be revealed.^31,32^ The proposed mechanism of Zosurabalpin is that it traps a substrate-bound conformation of the LPS transporter to inhibit its function.^31,32^ While this experimental drug appears to be specific to carbapenem-resistant *A. baumannii*, future research into ways to inhibit other Lpt proteins in a similar manner could prove impactful for developing new classes of antibiotics specific for Gram-negative bacteria. Peptide based drugs such as Zosurabalpin and antimicrobial peptides (AMP) have recently gained interest in the drug discovery process for targeting bacterial pathogens. However, there are key limitations to their use including but not limited to stability, weak antibacterial activity, toxicity, and high cost.^33^ Despite this, Zosurabalpin establishes that lipid mis-localization is a point of vulnerability that can be exploited to address the growing threat of multidrug-resistant Gram-negative bacteria.

### 3.2 Peptidoglycan Synthesis and Arrangement

Peptidoglycan (PG) synthesis is a carefully regulated process that is critical for maintaining cell shape and protecting against environmental stresses.^34,35^ Bacteria often rely on a minimal and conserved set of enzymes for peptidoglycan biosynthesis, making pathogens vulnerable to targeted disruptions of those enzymes. Well-known antibiotics like penicillin and vancomycin target PG biosynthesis.^36^ Penicillin acts as a suicide inhibitor of the transpeptidase involved in forming the cross-links while vancomycin sequesters the d-Ala-d-Ala transpeptidation substrate.^37^ More recently, amphiphilic and lipophilic cationic glycopeptides have been synthesized to overcome the inherent impenetrance of the outer membrane of Gram-negative bacteria to glycopeptides.^38^ Fosfomycin is another PG disrupting antibiotic that is particularly effective because it target an enzyme necessary for the early steps of PG biosynthesis.^39,40^

The enzyme MraY (phospho-*N*-acetylmuramyl-pentapeptide translocase) is a key player in PG biosynthesis. MraY catalyzes the crucial first step of transferring a PG precursor from a soluble UDP-linked form to a membrane-bound lipid carrier, thereby initiating cell wall assembly (Figure 2).^41,42^ Due to this role in maintaining bacterial cell integrity, MraY is considered a promising target for the development of new antibiotics.^41−46^ One antibiotic targeting MraY is tunicamycin, a natural product that binds to MraY’s cytoplasmic region.^45,47^ Tunicamycin inhibits the biosynthesis of peptidoglycan, but it has deleterious effects in eukaryotes due to its inhibition on eukaryotic protein *N*-glycosylation that leads to activation of the unfolded protein response.^50,51^ Moreover, tunicamycin and similar PG targeting antibiotics are not effective in Gram-negative bacteria because the outer membrane limits their cell penetrance.^49,50^ However, MraY is itself a challenging target, and some groups have looked away from small molecule inhibitors and instead considered developing monoclonal antibodies. There are other “overlooked” druggable sites on MraY that have gone unstudied which, in combination with its structural plasticity, could possibly present new avenues for targeted inhibition.^48^ MraY might be targeted in Gram-negative bacteria if drugs inhibiting the periplasmic end are used in conjunction with molecules that permeabilize the OM. Finally, advances into inhibitors of the other PG biosynthesis enzymes in the inner membrane such as MurG^52,53^ and MurJ^54,55^ show promise mostly for Gram-positive bacteria. Discovery of compounds or derivatives of inhibitors that also inhibit these enzymes in Gram-negative bacteria could prove fruitful for creating broad-spectrum based antibiotics.

### 3.3 Lipoprotein Transport—Differences within Gram-Negative Bacteria

Lipoprotein transport in Gram-negative bacteria is critical for the proper functioning and stability of the outer membrane as well as pathogenesis.^56−58^ Bacteria use the Lol system to transport lipoproteins from the inner membrane to the outer membrane. The system includes the periplasmic carrier protein LolA, the outer membrane protein LolB, and the inner-membrane ABC transport complex LolCDE (Figure 2).^59^ Recent advances have led to the development of Lolamicin, which specifically inhibits the LolCDE complex that is essential for the initial release of lipoproteins from the inner membrane.^60^ Targeting this pathway in bacteria that produce high numbers of surface lipoproteins can substantially compromise the bacterial envelope such that the bacteria are more susceptible to antibiotics and immune system clearance. Drugs like Lolamicin could work in conjunction with outer membrane permeabilization agents, which might be particularly useful for addressing the challenge of the double membrane barrier of Gram-negative bacteria.

## 4 Metabolite Synthesis and Trafficking

### 4.1 Isoprenoid Biosynthesis—MVA vs MEP Pathways

**Figure 3:**
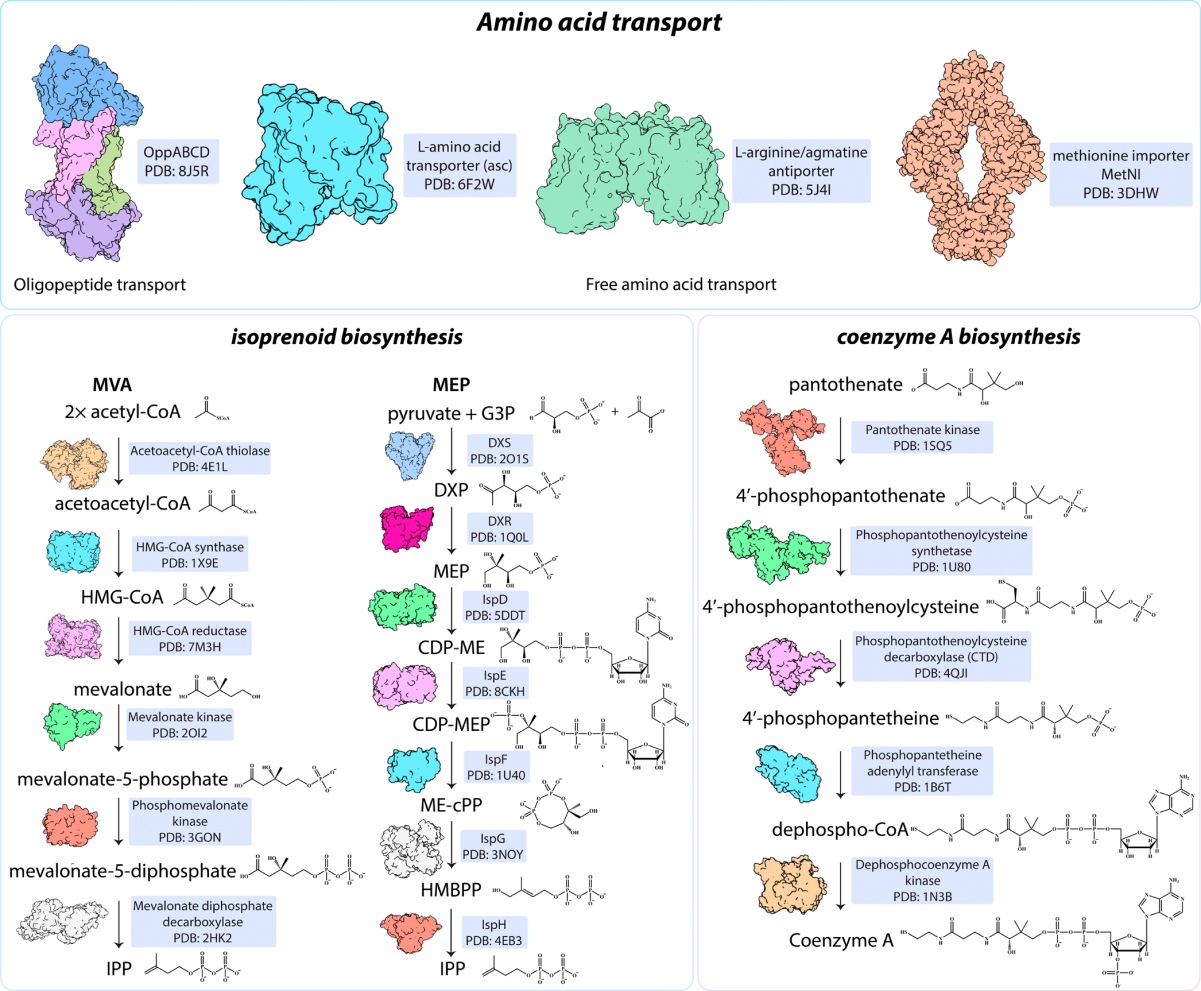
Essential metabolite synthesis and trafficking pathways with proteins that represent “opportunity” targets for new antimicrobial compound discovery.

Isoprenoid biosynthesis is essential to produce a wide range of metabolites that are necessary for bacterial cell survival as they form part of the cellular envelope, are involved in energy production, and are needed overall for cell maintenance.^61^ In most bacteria, the methylerythritol phosphate (MEP) pathway is the primary route for isoprenoid biosynthesis, and is distinct from the mevalonate (MVA, Figure 3) pathway used by eukaryotes and only a few bacteria.^62^ The specificity of the MEP pathway to bacteria makes it an attractive target for drug development. Enzymes in the MEP pathway are known to be essential, but the considerable biochemical and genetic plasticity in the pathway has impeded the design of inhibitors.^63^ The most studied inhibitor of the MEP pathway is fosmidomycin, an inhibitor of DXR (1-deoxy-D-xylulose 5-phosphate reductoisomerase) .^64^ There are also other reported inhibitors of enzymes in the MEP pathway, and we encourage interested readers to read more in the very detailed review by Allamand and colleagues.^65^ On the other hand, the MVA pathway is less common than the MEP pathway and fewer inhibitors target the bacterial version of this pathway. It commences with acetyl-coenzyme A and culminates with the formation of isopentenyl pyrophosphate (IPP), the precursor to many isoprenoids (Figure 3).^66^ IPP can further be modified to undecaprenyl phosphate (also known as C55-P or lipid-P), an essential molecule involved in the transport of polysaccharides and the lipid carrier for PG.

A key step in the MVA pathway is the conversion of hydroxymethyl coenzyme A (HMG-CoA) to mevalonate. This rate-limiting reaction is catalyzed by the enzyme HMG-CoA reductase (HMGR). Human HMGR is targeted by the statin class of drugs to control cholesterol biosynthesis,^67^ but the bacteria homologues are structurally and biochemically distinct and most make nonsteroidal isoprenoids, including C55-P.^68^ Statins display weak inhibition of bacterial HGMRs, thereby making it difficult to investigate the importance of the enzyme in bacterial strains.^68−77^ While difficult to do in many strains, genetic depletion of HMGR in *S. aureus* revealed that the enzyme is essential for cell proliferation.^78^ Additionally, a recent report of a phenyl sulfonamide inhibitor that targets the *Enterococcus faecalis* HMGR with single-digit IC_50_ values in vitro provides hope of the eventual delineation of the role that HMGR plays in bacteria.^79^ Future studies into the regulation of the mevalonate pathway in bacterial pathogens could yield several promising antimicrobial targets. Altogether, the inhibition of isoprenoid biosynthesis might be particularly impactful in bacteria, as the disruption of this pathway compromises their ability to maintain robust PG, among others.

### 4.2 Coenzyme A Biosynthesis

Coenzyme A (CoA) is an essential precursor for many metabolic pathways, including but not limited to, protein and fatty acid syntheses in bacteria.^80^ In Gram-negative bacteria, this pathway comprises five enzymatic steps (Figure 3). Recent drug developments have targeted enzymes in the CoA biosynthesis pathway such as pantothenate kinase (PanK) that catalyzes the initial step of converting pantothenate (vitamin B5) to CoA. This includes pantothenamides, which are inhibitors that mimic pantothenate and inhibit CoA biosynthesis to limit bacterial growth.^81−83^ These inhibitors might be particularly effective against bacteria that scavenge nutrients from the host, as disrupting CoA biosynthesis impairs their ability to metabolize host-derived fatty acids and other essential molecules. While pantothenamides are not the most potent (in *E. coli* and *K. pneumoniae*, the MIC values are 64 and 32 μg/mL respectively^84^), more can be done to design better analogs.

### 4.3 Amino Acid Transport

Amino acid synthesis and transport is necessary for the survival of many Gram-negative bacteria, especially those that lack the de novo synthesis pathway of amino acids (Figure 3). Thus, targeting bacterial peptide transport systems and free amino acid transporters presents a promising strategy. These systems are essential for bacterial survival as they facilitate the uptake of peptides and amino acids necessary for growth, virulence, and adaptation to host environments.^85−87^ Examples of amino acid and peptide transport systems in bacteria include the Opp (oligopeptide permease) and Dpp (dipeptide permease) systems.^88^ Targeting these systems could potentially impair the ability of these bacteria to thrive in hostile environments by limiting their ability to acquire amino acids.

Free amino acid transporters, like GltT, also represent promising drug targets in Gram-negative bacteria (Figure 3). These transporters differ from peptide transport systems in that they specifically import individual amino acids rather than peptides. Free amino acid transporters focus on single amino acids, with glutamate, serine/threonine, and cysteine as examples.^89−94^ An approach that disables such transport systems is particularly appealing because the transporters are often more specialized and selective, catering to the precise metabolic needs of the bacteria.

## 5 Methods and Case Studies of Bacterial Pathogens with Limited Metabolic Capabilities

### 5.1 Emerging Methods for Target Identification

Identifying novel drug targets in opportunistic bacterial pathogens requires sophisticated approaches that leverage advancements in multiple fields including genomics, proteomics, and computational biology. Genomic and proteomic approaches are at the forefront of this effort as they allow for the exploration of entire genetic and protein landscapes of a pathogen. Techniques like whole genome sequencing, comparative genomics, and transcriptomics provide detailed insights into essential genes and metabolic pathways that are critical for bacterial survival but absent in humans.^95,96^ Recently, researchers have identified unique bacterial enzymes and transport systems that are indispensable for survival in nutrient-limited environments.^97^ Proteomic analyses complements these efforts by quantifying protein expression under various conditions, helping to identify proteins that are upregulated during infection. Specific chemical proteomic approaches such as activity-based protein profiling,^98^ metabolic labeling,^99^ or thermal proteome profiling,^100^ have been shown to be very useful in not only identifying new targets, but also understanding new modes of inhibition and host–pathogen interactions.

In parallel, computational and bioinformatic tools have become invaluable in predicting new targets for drug development. These tools utilize vast data sets generated by genomic and proteomic studies to model bacterial metabolic pathways and identify bottlenecks that can be therapeutically exploited. Machine learning algorithms are increasingly used to predict the essentiality of genes based on their evolutionary conservation, structural properties, interaction networks, and functional roles.^101,102^ Additionally, molecular docking simulations and virtual screening methods allow for the rapid testing of potential inhibitors against identified targets, which has accelerated the drug discovery process.^103−105^ These emerging methods are particularly valuable for targeting opportunistic pathogens, as they identify and exploit vulnerabilities that may not be apparent through traditional experimental approaches and allow for the study of bacteria that are challenging to culture.

### 5.2 Case Studies

#### 5.2.1 Targeting Cellular Envelope Construction in *Treponema pallidum* and *Borrelia burgdorferi*

*Treponema pallidum* (*T. pallidum*), the causative agent of syphilis, and *Borrelia burgdorferi* (*B. burgdorferi*), responsible for Lyme disease, are two spirochetes with limited proteomes that rely heavily on their host for nutrients.^106^ A key example of this lies in bacterial cell envelope construction. Our knowledge of how these bacteria construct their cellular envelope is limited. Current understanding of this process from bacteria in general is mainly from studies done on a few model organisms such as *E. coli*. However, some pathogens, like *B. burgdorferi* and *T. pallidum* deviate from these models by incorporating host lipids into their membranes. Both pathogens do not perform the de novo synthesis of long chain fatty acids and rely on host-derived lipids, making enzymes involved in fatty acid acquisition and utilization of particular interest. The two pathogens also lack lipopolysaccharide (LPS) and instead have a wide range of essential outer membrane proteins (OMPs) and surface lipoproteins, many of which have recently been identified as potential vaccine targets.^107,108^ An example is TprK in *T. pallidum*, which is garnering interest due to its role in host immune system evasion and antigenic variation.^109^ Similarly, *B. burgdorferi* surface lipoproteins OspA, OspC, and VlsE are critical for transmission, colonization, and immune system interactions.^110^ More recently, these proteins have been explored as vaccine candidates and drug targets.^111^ Elucidating the mechanisms that govern cellular envelope construction in these unique spirochetes could open a new avenue for antimicrobial discovery.

#### 5.2.2 Intracellular Pathogens

*Chlamydia trachomatis* (*C. trachomatis*), *Neisseria gonorrheae* (*N. gonorrheae*), and *Legionella pneumophila* (*L. pneumophila*) are the causative agents of chlamydia, gonorrheae, and Legionnaire’s disease, respectively. These are intracellular pathogens that hijack host machinery through many modes and are extremely hard to culture, study, and inhibit.^112−114^ These pathogens also have limited proteomes, leading to increased reliance on host-derived resources. *C. trachomatis* has approximately 900 protein-coding genes (D/UW-3/Cx strain of *C. trachomatis* contains 935 genes).^115^*N. gonorrheae* encodes ∼2,000 (strain ATCC 700825/FA 1090 contains 2,106 proteins),^116^ and *L. pneumophila* has ∼3,000 (*L. pneumophila* subsp. pneumophila (strain Philadelphia 1/ATCC 33152/DSM 7513 has 2,930 proteins).^117^ In contrast, a model organism like *E. coli* possesses over 4,000 genes. Bioinformatic analyses can be used to identify critical proteins as potential drug targets in these intracellular pathogens. There are a few examples shown in *C. trachomatis*. One example includes the major outer membrane protein (MOMP), which plays a role in nutrient uptake and immune evasion.^118^ A second example is the Type III secretion system (T3SS) effector protein, Tarp (Translocated actin-recruiting phosphoprotein), that is essential for the pathogen’s ability to manipulate host cell actin dynamics and also facilitating entry and replication.^119^ Inhibiting proteins like these could disrupt the pathogen’s ability to hijack host cell processes.

*N. gonorrheae* is notorious for its rapid development of antibiotic resistance. Much research is being conducted to understand its biology as well as the development of drugs that evade resistance. Recently, key proteins involved in iron acquisition, such as TbpA (Transferrin-binding protein A)^120^ and FetA (ferric enterobactin receptor)^121^ were identified and characterized as potential “druggable” targets. These proteins are crucial for pathogen survival in iron-limiting conditions which is a common challenge during infection within the host environment. Targeting these systems could weaken the bacterium’s innate defense mechanisms, rendering it more susceptible to treatment.

*L. pneumophila* has a complex intracellular lifestyle that involves strategically manipulating host cell processes for its advantage.^114^ Bioinformatics and biochemical tools have identified several essential genes involved in lipid metabolism, such as LppA (*L. pneumophila* lipase) and enzymes of the Dot/Icm Type IV secretion system, which are critical for the bacterium’s ability to secrete virulence factors into host cells.^122,123^ Disrupting these systems could prevent the bacterium from establishing a infection and replicating within host cells.

Another vulnerable mechanism that could be potentially explored lies with host derived lipids. Because they lack de novo synthesis for many lipids, these bacteria depend heavily on host lipids for critical functions, including membrane synthesis and energy production. There are currently no specific examples of how these lipid transport pathways could be targeted in bacteria primarily because of the lack of key biochemical and structural data. Sometimes the identities of these transporters are not even known. However, there are drugs that have targeted lipid metabolism in parasites such as *Plasmodium falciparum.*(124)*C. trachomatis* is known to hijack host lipid pathways to inclusion membrane essential for its intracellular replication.^125^ Similarly, *L. pneumophila* exploits host lipids within its replicative vacuole, ensuring its proliferation.^126^*N. gonorrheae* also mimics host lipids to facilitate its infection process and contribute to its virulence.^127^ The dependence on host lipids underscores a key adaptation mechanism that allows the pathogens to thrive despite their reduced biosynthetic capacity. Strategies to exploit this dependence could include inhibiting host lipid transfer to pathogens, disrupting bacterial lipid utilization pathways, or modulating host lipid metabolism to limit bacterial access and effectively impair their ability to proliferate and cause disease. Along with these strategies, more recent studies have focused on host-directed therapies for eliminating obligate intracellular bacteria and we encourage readers to read more in the review by Kaufmann, Stefan., et al.^128^

## 6 Call-to-Action

The fight against AMR requires an integrated approach that shifts away from traditional methods. The impact of scientific advancements when they are translated into clinical practice, are heightened by a full embrace of the community. When thinking about bringing these therapies from the laboratory to the bedside, it is equally important to engage with communities to ensure that these new treatments are accessible and used appropriately.

The impact of natural phenomena such as climate change and other natural disasters on antibiotic resistance is an emerging concern that continues to disproportionately affect marginalized communities and under-resourced countries. Environmental changes can (and have) exacerbated the spread of resistant bacteria through increased temperature and changes in water sources. This can alter the prevalence and distribution of pathogens. These challenges are compounded in marginalized communities, where access to clean water, healthcare, and education about antibiotic resistance is often limited. This is directly seen with the Flint, Michigan water crisis. Legionaries disease continues to be on the rise due to contaminated water systems where the intracellular pathogen, *L. pneumophilia*, tends to reside causing a large increase in infections.^129^

Addressing these issues begins with a global approach that includes improving sanitation, access to effective healthcare, and promoting responsible antibiotic use. By focusing on the environmental and social factors of antibiotic resistance, we can begin to develop strategies that protect the most vulnerable populations, while reducing the global burden of AMR infections. Efforts can include educational initiatives that inform the public about the importance of proper antibiotic use and the consequences of self-medication. Additionally, collaborating with global health organizations, policymakers, and stakeholders that include those with financial resources, is vital to ensure that new treatments reach marginalized communities where the burden of antibiotic resistance is often highest. By integrating basic and translational research with clinical practice and community engagement, we can create a more comprehensive and effective response to the growing threat of AMR.

## 7 Conclusion

In this outlook, we chose to highlight what we deem as “opportunity targets”, which represent essential pathways with potentially druggable proteins for which resistance mechanisms might be difficult or slow to arise. We also describe recently reported experimental antibiotics that target some of these proteins and discuss new methods to uncover important druggable pathways. Finally, we discuss to global impact of AMR and include a call-to-action of how stakeholders can coordinate to abate the crisis. We do note that many of the opportunity targets are unique to the strains we focus on and any antibiotics targeting them would be narrow in scope. However, because these would be new targets, they might be less prone to inactivation via existing mechanisms. Moreover, putative narrow spectrum inhibitors would leave important commensal bacterial communities such as the gut microbiome unaffected. In summary, we hope this Outlook calls attention to potential solutions for the AMR crisis.
